# A Prospective Comparative Study of Health Inequalities and the Epidemiology of Stroke in French Guiana and Dijon, France

**DOI:** 10.3389/fpubh.2022.849036

**Published:** 2022-05-13

**Authors:** Devi Rita Rochemont, Emmanuelle Mimeau, Caroline Misslin, Martine Papaix-Puech, Bertrand de Toffol, Nadia Sabbah, Emmanuel Delmas, Yannick Bejot, Isabelle Fournel, Mathieu Nacher

**Affiliations:** ^1^CIC Inserm 1424, Centre d'investigation Clinique, Centre Hospitalier de Cayenne, Cayenne, French Guiana; ^2^Service d'accueil des Urgences, Centre Hospitalier de Cayenne, Cayenne, French Guiana; ^3^Service de Médecine, Centre Hospitalier de l'Ouest Guyanais, Saint-Laurent-du-Maroni, French Guiana; ^4^Service des Urgences, Centre Hospitalier de Kourou, Kourou, French Guiana; ^5^Service de Neurologie, Centre Hospitalier de Cayenne, Cayenne, French Guiana; ^6^Service de Diabétologie, Centre Hospitalier de Cayenne, Cayenne, French Guiana; ^7^Centre d'investigation Clinique – Épidémiologie Clinique, CIC Inserm 1432, Centre d'investigation Clinique, CHRU de Dijon, Dijon, France; ^8^Registre des AVC de Dijon, EA7460, Service de Neurologie, Université de Bourgogne, CHRU Dijon, Dijon, France; ^9^UFR des Sciences de Santé, Université de Bourgogne, Dijon, France; ^10^Département Formation Recherche (DFR) Santé, Université de Guyane, Cayenne, French Guiana

**Keywords:** ischemic stroke, risk factors, thrombolysis, health inequalities, epidemiology, French Guiana

## Abstract

**Background:**

In French Guiana poverty is widespread and specialized care is lacking. We aimed to compare strokes between precarious and non-precarious patients within French Guiana and to compare the epidemiology of ischemic strokes and their outcomes between French Guiana and mainland France.

**Methods:**

A multicenter prospective cohort examined the influence of social inequalities on stroke characteristics. Consecutive patients aged > 18 years admitted for an acute ischemic stroke, confirmed by neuroimaging were eligible. Exclusion criteria were a history of symptomatic stroke, presence of other short-term life-threatening diseases and inability to contact patients by telephone during follow-up. Social deprivation was measured using the EPICES score, which is based on a multidimensional questionnaire.

**Results:**

Overall, 652 patients with ischemic stroke were included. The patients in French Guiana were 7 years younger, were more frequently male, of sub-Saharan ancestry, they had a low level of education, and were more often precarious (67.7%) than the patients included in Dijon (39.2%). The origin of the ischemic stroke was predominantly lacunar for patients included in French Guiana and cardioembolic for patients included in Dijon, with greater severity for patients included in Dijon. The proportion of patients with known pre-stroke hypertension, diabetes, or a history of Transient Ischemic Accident was greater in French Guiana than in Dijon. In contrast, hypercholesterolemia, atrial fibrillation, and history of Myocardial Infarction were more frequently found in patients included in Dijon than in patients included in French Guiana. Fibrinolysis was less frequent in French Guiana than in Dijon, 24% of patients arriving early enough receiving thrombolysis in French Guiana vs. 45% in Dijon, *P* < 0.0001. However, after adjustment for patient characteristics, the effect of the center on the use of fibrinolysis disappeared. When comparing precarious and non-precarious patients within French Guiana, the main difference was the younger age and the lower mortality of precarious patients—notably immigrants.

**Conclusion:**

Precariousness was widespread in French Guiana. Within French Guiana, despite a younger age among foreigners than French patients, the risk factors, mechanisms, and outcomes were homogenous across socioeconomic strata. The observed differences between the two contrasted French territories suggested that, beyond health inequalities, the epidemiology of cardiovascular risk factors may differ between French Guiana and mainland France.

## Introduction

Stroke represents a major cause of death, disability and dementia worldwide (1). It has been known for decades that the incidence and prognosis of stroke are worse in low and middle income countries relative to high income countries, a difference that has been increasing over time (1). Furthermore, within high income countries, there are also major differences in incidence and prognosis of stroke between the more socially disadvantaged groups relative to less disadvantaged (2). Although it is difficult to disentangle ethnicity and social factors, it has been suspected that ethnicities of African ancestry were more at risk for stroke; they also had a poorer prognosis. Whereas, some studies suggested that 39% of the excess mortality in Blacks was due to lower socioeconomic status, others, controlling for risk factors and health insurance level, found that the differences between blacks and non-blacks were in fact explained by the higher prevalence of standard cardiovascular risk factors in blacks relative to non-blacks (3).

French Guiana is a multiethnic Amazonian territory with populations from African, European, and Asian ancestry. It is also a territory of great social contrast: It has the highest GDP per capita in Latin America, while 29% of the total population, and nearly half of adults, consists of foreigners with great social vulnerabilities (4). The incidence of patients hospitalized for ischemic stroke and hemorrhagic strokes in French Guiana is the highest of all French territories (5, 6). The overall standardized incidence of stroke was 297 per 100 000 in 2017 (7). We have previously shown that social inequalities of health were present for nearly all major pathologies –HIV, Cancer, Preterm birth, terminal renal failure- and that precarious populations often renounced care (8–13). Despite being a French territory, French Guiana has a shortage of medical and paramedical staff, and a lack of specialized structures. Hence in 2018, there were an estimated 652 doctors and 2.4 neurologists per 100,000 inhabitants (14). The social differences in incidence, severity, and prognosis of strokes are possibly the result of greater frequency of risk factors, problems in accessing care, and the quality of emergency care and rehabilitation therapy. At a time when progress on the control of risk factors and improvement of emergency care of strokes have led to significant progress, the most socially vulnerable segments of the population may not benefit equally from these advances. Between 2011 and 2015 INDIA a prospective study was conducted in French Guiana and in Dijon, France. Because of the sociocultural complexity of French Guiana (4, 15) and its lack of specialized care (16), we hypothesized that precariousness would be frequent in our cohort, and that it may influence presentation and outcomes. Our main objectives were to compare the epidemiology of ischemic strokes and their outcomes between French Guiana and mainland France, and to compare strokes between precarious and non-precarious patients within French Guiana; the main outcomes studied were risk factors, case fatality, stroke mechanism, treatment of stroke and outcomes were both compared between French Guiana and Dijon, and within French Guiana between the precarious and non-precarious.

## Methods

### Study Design

**Figure 1:**
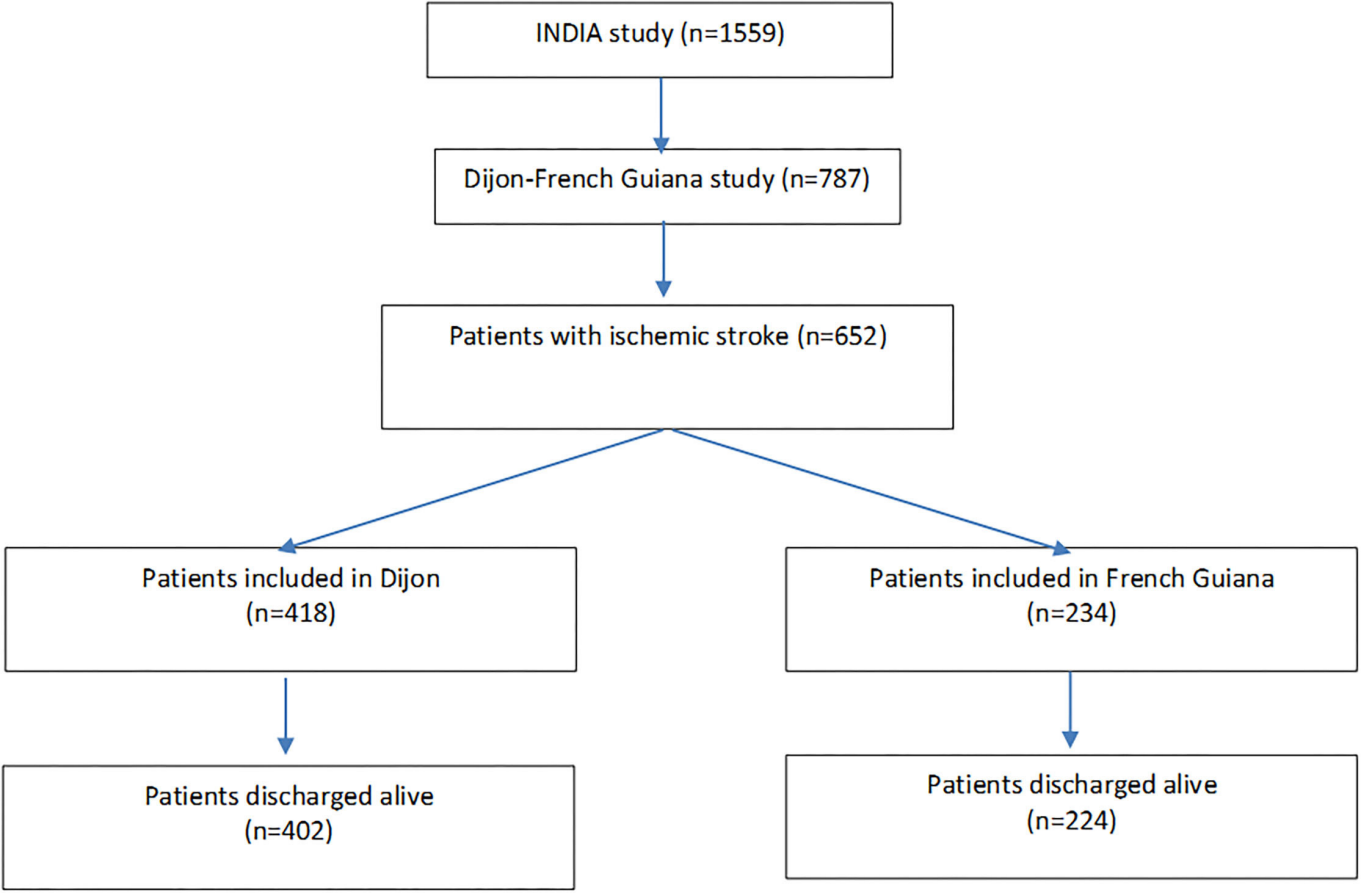
Flow chart of patients included in Dijon and French Guiana.

The French multicenter INDIA prospective cohort study (INégalités sociales et pronostic des accidents vasculaires cérébraux à Dijon et en Antilles-Guyane) was designed to examine the influence of social inequalities on stroke characteristics and prognosis and was previously described (5). Consecutive patients aged > 18 years admitted for an acute ischemic stroke, confirmed by neuroimaging, and who were able to be interviewed either personally or *via* a next of kin were eligible. Exclusion criteria were a history of symptomatic stroke, presence of other short-term life-threatening diseases and inability to contact patients (or support persons) by telephone during follow-up. Between June 2011 and October 2014, 1,573 patients were recruited in three neurology departments of university hospitals (Dijon, Burgundy; Fort-de-France, Martinique, Pointe à Pitre, and Guadeloupe) and emergency or medicine departments of three hospitals in French Guiana (Cayenne, Saint-Laurent du Maroni, Kourou). Among them, patients with subarachnoid hemorrhage and hemorrhagic stroke were excluded. For the present study, only participants with ischemic stroke included in French Guiana and Dijon were included (*n* = 652). Figure 1 shows the study flowchart. In French Guiana most patients were included in Cayenne (Cayenne 244 patients, Saint Laurent du Maroni 37 patients, and Kourou 17 patients). In French Guiana, there was no stroke unit so patients were admitted in normal medical wards.

According to French law relative to observational studies, all patients received written information about the study but signed consent was not required. The study protocol was approved by the Burgundy Ethics Committee (CPP Est 1, 16 May 2010) and the National Commission for Data Processing and Civil Liberties (15 April 2011).

### Collected Data

Demographic data, preexisting conditions, pre-stroke mRS, admission NIHSS score categorizing stroke severity upon patient arrival and mechanism of stroke were collected at the time of inclusion. mRs and NIHSS score were also collected after discharge. Anamnestic information included medications, history of ischemic vascular diseases, known and suspected cardiovascular risk factors. Patients or patient kin were interviewed on the patient's socio economic status including geographic origin of parents or ancestors, marital status, employment status, education, housing conditions, wealth indicators. They were also interviewed on the patient's healthcare access including the time to reach the nearest primary care physician, the nearest hospital, health insurance, the number of consultations with a primary care physician, a dentist or a specialist within the previous year, colorectal cancer screening, previous hospitalization ≥24 h in the 12 preceding months. Stroke therapies (including thrombolysis), healthcare trajectories, the duration of hospital stay, complementary examinations, and vital status at hospital discharge were also reported. The follow-up phone calls took place at 1, 6, and 12 months.

Social deprivation was measured at the individual level using the Evaluation de la Précarité et des Inégalités de santé dans les Centres d'Examen de Santé (EPICES) score, which ranges from 0 (no deprivation) to 100 (Maximum deprivation) (17, 18). The EPICES score is based on a multidimensional questionnaire comprising 11 items exploring socioeconomic conditions, leisure activities and family/social support and it can be used to assess precariousness and social deprivation between contrasted areas (5, 6). Patients were defined as precarious if the EPICES score was >30.

### Statistical Analysis

Descriptive results were expressed as percentages for categorical variables and as means +/- SD or medians [IQR] for continuous variables, as appropriate. Qualitative variables were compared with the Chi2 test, and quantitative variables with Student's *t*-test or non-parametric tests, as appropriate. Statistical analyses were performed with STATA 12 software (College Station, Texas). The statistical significance was set at 5%. In addition, unconditional logistic regression was used to adjust for potential confounders.

### Ethics

The cohort was approved by the French Regulatory authorities CNIL (Commission Nationale de l'Informatique et des Libertés, autorisation n°911040) and the CPP Est 1/AFSSAPS (Comité de protection des personnes Est 1, Agence National de Sécurité du médicament et des Produits de Santé, autorisation n°2010/40 – AFSSAPS n°2010-A00940-39).

## Results

### Comparisons Between French Guiana and Dijon

Figure 1 shows the flowchart of the INDIA study and the French Guiana-Dijon analysis. The patients included in French Guiana were nearly 7 years younger, were more frequently male, of sub-Saharan ancestry, with a level of education below the baccalaureate, and more often precarious than the patients included in Dijon—67.7 vs. 39.4%. Patients included in French Guiana were also more often born in another country than patients included elsewhere (52 vs. 7.3%, *p* < 0.0001). Supplementary Table 1 shows the comparisons of socioeconomic characteristics and health care use between patients included in French Guiana and patients included in Dijon, France.

Access time to the nearest GP or hospital was greater for patients included in French Guiana. Patients included in French Guiana were less likely to have health insurance, but more likely to rely on welfare pensions or insurance [Revenu Minimum d'Insertion, Revenu de Solidarité Active, minimum vieillesse, Couverture Maladie Universelle complémentaire (CMUc), Aide Médicale Etat] than patients included in Dijon, with less recourse to doctors (general practitioner and specialist) and dentists (Supplementary Table 1).

#### Stroke Mechanism

The origin of the ischemic stroke was predominantly lacunar for patients included in French Guiana and cardioembolic for patients included in Dijon, with a greater severity for patients included in Dijon (Table 1). Before their first ischemic stroke, patients included in French Guiana were less often on platelet antiaggregants or anti-coagulants than patients included in Dijon (Table 1).

**Table 1:** Stroke presentation, risk factors, and care comparisons between French Guiana and Dijon.

	**Dijon**		**French Guiana**		***P***
	***N* (418)**	**%**	***N* (234)**	**%**	
**Stroke classification**					
**TOAST classification**					<0.0001
Incomplete	23	5.5	57	24.4	
Large arteries	99	23.7	38	16.2	
Cardioembolic	157	37.6	28	12.0	
Other cause	139	33.2	111	47.4	
**NIHSS score at admission**					0.0013
0–3	115	27.5	75	33.8	
4–9	123	29.4	84	37.8	
≥10	180	43.1	63	28.4	
**Clinical characteristics**					
BMI					<0.0001
<20	22	5.3	11	4.7	
[20–25]	122	29.2	60	25.6	
[25–30]	166	39.7	68	29.1	
≥30	94	22.5	41	17.5	
Missing	14	3.3	54	23.1	
Known hypertension before the stroke	251	60.1	164	70.4	0.008
Diabetes	77	18.4	67	28.8	0.002
Known hypercholesterolemia	131	31.3	42	18.2	0.0003
Heart failure	36	8.6	12	5.2	0.109
Atrial fibrillation	134	32.1	17	7.4	<0.0001
Previous TIA	24	5.8	33	14.1	0.0003
History of MI	28	6.7	5	2.1	0.011
Alcohol consumption	269	64.5	132	57.6	0.085
Current smoking	82	19.6	53	22.8	0.345
**Therapeutic characteristics**					
Anti-platelet agents	115	27.5	34	14.5	0.0002
Anticoagulants	42	10.0	10	4.3	0.010
Anti-hypertensives	247	59.2	120	51.7	0.064
IV fibrinolysis	166	39.7	32	13.7	<0.0001
IV fibrinolysis among 410 patients who arrived within 4.5 h of stroke	127	45.0	31	24.2	<0.0001

#### Frequency of Thrombolysis

In bivariate analysis, the use of intravenous fibrinolysis was significantly less frequent in patients included in French Guiana than in patients included in Dijon; This remained true when the analysis was restricted to patients who arrived within a time frame compatible with fibrinolysis with only 24% of patients arriving early enough treated by thrombolysis in French Guiana vs. 45% in Dijon, *P* < 0.0001 (Table 1). However, when the analysis of patients who arrived within a time frame compatible with fibrinolysis adjusted for patient characteristics, the effect of the center on the use of fibrinolysis disappeared (Table 2).

**Table 2:** Factors associated with thrombolysis.

	**Univariate (*****n*** **=** **317)**			**Full model (*****n*** **=** **317)**			**Step by step (*****n*** **=** **317)**		
	**Crude OR**	**IC 95%**	***P***	**Adjusted OR**	**IC 95%**	***P***	**Adjusted OR**	**IC 95%**	***P***
French Guiana vs. Dijon	0.52	0.31–0.88	0.01	0.80	0.39–1.64	0.55	0.78	0.39–1.56	0.48
Age ≥ 65 vs. <65 years	0.90	0.56–1.42	0.65	0.67	0.35–1.27	0.22	0.63	0.34–1.15	0.13
Woman vs. man	1.14	0.73–1.78	0.55	0.66	0.37–1.16	0.15	0.64	0.37–1.13	0.12
High school degree	0.64	0.37–1.10	0.11	0.62	0.32–1.21	0.16	0.64	0.33–1.23	0.18
BMI			0.006			0.002			
<20	1.12	0.40–3.07	0.82	1.24	0.36–4.19	0.72	1.26	0.38–4.23	0.7
[20–25]	REF			REF			REF		
[25–30]	0.45	0.26–0.79	0.005	0.39	0.20–0.75	0.005	0.38	0.20–0.73	0.004
≥30	0.47	0.24–0.92	0.02	0.39	0.17–0.87	0.02	0.38	0.17–0.83	0.01
Missing	0.22	0.08–0.63	0.005	0.14	0.04–0.47	0.001	0.14	0.04–0.47	0.001
Known hypertension before the stroke	0.69	0.44–1.08	0.10	0.78	0.43–1.44	0.44			
Diabetes	0.62	0.34–1.14	0.12	0.59	0.28–1.22	0.15	0.56	0.27–1.15	0.11
Hypercholesterolemia	1.67	1.00–2.79	0.04	2.77	1.44–5.34	0.002	2.65	1.39–5.03	0.003
Atrial fibrillation	1.43	0.87–2.36	0.15	1.03	0.50–2.10	0.93			
Previous TIA	0.51	0.23–1.13	0.10	0.41	0.16–1.03	0.05	0.41	0.16–1.04	0.06
**TOAST classification**			0.002			0.02			0.01
Lacunes	0.12	0.04–0.38	0.0003	0.17	0.04–0.62	0.008	0.16	0.04–0.57	0.005
Large arteries	0.59	0.32–1.08	0.09	0.54	0.25–1.19	0.13	0.55	0.27–1.09	0.09
Cardioembolic	REF			REF			REF		
Other cause	0.85	0.50–1.45	0.56	0.90	0.43–1.89	0.79	0.91	0.47–1.75	0.78
**NIHSS score at admission**			<0.0001			<0.0001			<0.0001
0–3	REF			REF			REF		
4–9	5.75	2.80–11.77	<0.0001	8.01	3.56–18.05	<0.0001	8.13	3.61–18.30	<0.0001
≥10	6.53	3.28–13.00	<0.0001	7.65	3.51–16.65	<0.0001	7.72	3.55–16.80	<0.0001

#### Comorbidities

In terms of comorbidities, the proportion of patients with known pre-stroke hypertension, diabetes, or a history of Transient Ischemic Accident was greater in patients included in French Guiana than in patients included in Dijon (Table 1). In contrast, hypercholesterolemia, atrial fibrillation, and history of Myocardial Infarction were more frequently found in patients included in Dijon than in patients included in French Guiana (Table 1).

Concerning the Body Mass Index (BMI), the proportion of missing data on this variable was significantly higher in patients included in French Guiana. In contrast, for patients with available data, the mean BMI did not differ significantly between patients included in French Guiana and patients included in Dijon (Table 1).

#### Destination at Discharge

The patients included in French Guiana were more likely to be discharged toward home than the patients included in Dijon. The frequency of patients receiving rehabilitation did not differ significantly between Dijon and French Guiana, but rehabilitation appeared to be more quickly available in French Guiana than in Dijon, with the majority of patients included in Dijon being transferred to other wards upon discharge, whereas the majority of patients included in French Guiana returned home.

#### Prescription of Physical Therapy for Patients Discharged Home

The differences in physical therapy prescription observed between Dijon and French Guiana were explained by differences in patient characteristics; after adjustment for patient characteristics, the prescription of physical therapy when patients returned home was no longer associated with the inclusion center. There was no significant difference between the centers in the prescription of speech therapy sessions.

### Comparisons Between Socially Precarious and Non-precarious Patients Within French Guiana

Table 3 shows the comparisons between socially precarious and non-precarious ischemic stroke patients and between foreign-born and French-born patients for patients in French Guiana.

**Table 3:** Comparisons between precarious and non-precarious patients within French Guiana.

	Socially precarious^*^	Socially non-precarious^*^	***p***	**Foreign-born**	**French-born**	***p***
	**(Epices score > 30)**	**(Epices score <31)**		**(*n* = 126)**	**(*n* = 108)**	
	**(*n* = 159)**	**(*n* = 74)**				
**Vascular risk factors**						
Mean Age (SD)	61.56 years	63.92 years	0.2	60.6 years	64.3 years	0.04
Hypertension *N* (%)	111 (70.25)	52(70.27)	0.99	94 (75.2)	70 (64.8)	0.08
Diabetes *N* (%)	42 (26.58)	24 (32.43)	0.36	35 (28)	32 (29.63)	0.78
Body mass index Mean (SD)	26.96 (5.18)	26.37 (4.09)	0.46	26.54 (5.26)	27.045 (4.40)	0.49
Hypercholesterolemia *N* (%)	28 (17.72)	14 (19.44)	0.75	19 (15.2)	23 (21.7)	0.2
Cardiac failure *N* (%)	9 (5.73)	3 (4.11)	0.61	4 (3.23)	8 (7.48)	0.15
Atrial fibrillation *N* (%)	12 (7.69)	5(6.94)	0.84	1 (0.65)	9 (8.49)	0.57
Alcohol (+3 glasses/day) *N* (%)	15 (9.74)	7 (9.46)	0.95	10 (8.13)	12(11.32)	0.41
Tobacco *N* (%)	60 (37.97)	29 (39.19)	0.86	46 (36.8)	44 (40.74)	0.54
Depression *N* (%)	11 (7.05)	5 (6.85)	0.96	5 (4.07)	11 (10.28)	0.06
Oral contraceptive *N* (%)(women only)	5 (8.62)	2 (6.67)	0.75	3 (10.64)	1 (4.88)	0.32
Substitution Hormonotherapy substitutive *N* (%)(women only)	4 (6.90)	0 (0)	0.14	47 (6.38)	41 (3.44)	0.59
**Prior vascular history**						
Transient ischemic accident *N* (%)	21 (13.21)	12 (16.22)	0.54	13 (10.32)	20 (18.52)	0.07
Myocardial infarction *N* (%)	4 (2.52)	1 (1.35)	0.57	2 (1.59)	3 (2.78)	0.53
Lower limb arteriopathy *N* (%)	8 (5.03)	5 (6.76)	0.59	2 (1.59)	11 (10.19)	0.0042
*Angina pectoris N* (%)	2 (1.26)	4 (5.4)	0.06	2 (1.6)	4 (3.7)	0.31
Vascular surgery *N* (%)	4 (2.52)	3 (4.05)	0.52	0 (0)	7 (6.48)	0.0037
Angioplasty *N* (%)	4 (2.52)	2 (2.70)	0.93	1 (0.79)	5 (4.63)	0.06
**Prior vascular history in the family**						
Myocardial infarction (fam) *N* (%)	8 (8.2)	5 (7.7)	0.91	4 (5.25)	9 (10.11)	0.25
Stroke (fam) *N* (%)	27 (22.69)	14 (21.21)	0.82	16 (17.2)	26 (27.96)	0.08
Diabetes (fam) *N* (%)	52 (42.63)	24 (36.36)	0.40	36 (38.71)	40 (41.67)	0.68
Hypertension (fam) *N* (%)	79 (66.39)	36 (59.01)	0.33	62 (68.13)	54 (60)	0.25
**Treatment before stroke**						
Platelet anti-agregant *N* (%)	24 (15.09)	9 (12.16)	0.55	13 (10.32)	21 (19.44)	0.048
Anti-coagulant *N* (%)	6 (3.8)	4 (5.48)	0.56	4 (3.3)	6 (5.6)	0.37
Anti-hypertensive treatment *N* (%)	79 (50.32)	40 (54.05)	0.6	62 (50.0)	58 (53.7)	0.57
Oral anti-diabetic *N* (%)	26 (16.46)	18 (24.66)	0.14	22 (17.6)	22 (20.56)	0.56
Statin *N* (%)	22 (13.84)	9 (12.33)	0.75	12 (9.53)	19 (17.76)	0.065
Insulin *N* (%)	17 (10.69)	4 (5.48)	0.2	10 (7.9)	11 (10.28)	0.53
Psychotropic treatment *N* (%)	5 (3.38)	3 (4.11)	0.90	6 (4.8)	3 (2.8)	0.44
Oral complements *N* (%)	4 (2.52)	0 (0)	0.17	3 (2.38)	1 (0.9)	0.4
**Handicap prior to stroke**						
Rankin no symptom *N* (%)	131 (82.91)	68 (91.89)	0.068	108 (86.4)	92 (85.19)	0.79
Rankin minimal symptom (1–2/6) *N* (%)	13 (8.22)	1 (1.35)	0.04	8 (6.4)	6 (5.56)	0.79
Rankin moderate to severe symptom (3–5/6) *N* (%)	14 (8.86)	5 (6.76)	0.59	9 (7.20)	10 (9.26)	0.57
Barthel complete autonomy *N* (%)	146 (92.41)	72 (98.63)	0.056	117 (93.6)	102 (95.33)	0.57
**Delay of care**						
Stroke –> Admission (mean in hours/iqr)	11.62 (1.23–13.62)	8.43 (1.52–6.63)	0.26	12.4 (1.22–11.75)	8.68 (1.53–8.34)	0.17
Admission –>Thrombolysis (mean in hours/iqr)	2.27 (1.47–2.68)	2.11 (1.5–2.85)	0.6869	2.59 (1.65–3.34)	1.75 (1.21–2.23)	0.037
Stroke–> Thrombolysis (mean in hours/iqr)	3.41 (2.92–4.33)	4.11 (2.5–4.5)	0.3501	4.03 (3.08–4.46)	3.35 (2.33–4.33)	0.36
Thrombolysis performed *N* (%)	159 (11.95)	74 (17.57)	0.25	126 (12.7)	108 (14.81)	0.64
**Explorations realized**						
Scanner	133 (83.6)	59 (79.73)	0.46	103 (81.74)	90 (83.33)	0.75
1st MRI	95 (59.75)	56 (75.68)	0.018	71 (56.35)	81 (75)	0.0029
2nd MRI	4 (2.52)	2 (2.70)	0.93	4 (3.17)	2 (1.85)	0.52
Cervical and intracranial angioscanner	94 (59.12)	44 (59.46)	0.96	76 (60.32)	63 (58.33)	0.76
Supraaortic and transcranial doppler and ultrasonography	89 (55.97)	48 (64.86)	0.1	61 (48.4)	77 (71.3)	0.0004
EKG	158 (99.37)	74 (100)	0.49	125 (99.21)	108 (100)	0.35
Holter	75 (47.3)	35 (47.17)	0.99	59 (46.83)	51 (47.22)	0.95
Transthoracic Ultrasonography (TTU)	132 (83.02)	57 (77.03)	0.28	104 (82.54)	86 (79.63)	0.57
Transoesophageal ultrasonography (TU)	11 (6.92)	6 (8.11)	0.75	8 (6.35)	9 (8.33)	0.56
**Type of stroke**						
Stroke severity: NIH > 10 (*N*/%)	43 (28.48)	14 (20)	0.18	33 (27.97)	24 (23.08)	0.41
Atheroma without supra-aortic stenosis	57 (35.85)	28 (37.84)	0.77	43 (34.13)	43 (39.81)	0.37
Intracranial atheroma	14 (8.81)	7 (8.81)	0.86	10 (7.94)	10 (9.26)	0.72
Sequelae of prior systematized stroke on imaging	10 (6.29)	10 (13.51)	0.067	7 (5.56)	13 (12.04)	0.077
Sequellae of prior lacunae on imaging	37 (23.27)	23 (31.08)	0.2	17 (24.60)	18 (26.85)	0.69
**Exit mode**						
Mean hospitalization duration (days)	14.18	17.62	0.36	13.83	16.97	0.37
Stroke severity at discharge: NIH > 10 (*N*/%)	17 (18.48)	6 (14.63)	0.59	16 (21.05)	7 (12.07)	0.17
OR after adjusting for age	OR: 1.36	[IC:0.48–3.75]	0.56	OR: 1.97	[IC:0.75–5.18]	0.17
Aphasia	18 (11.61)	8 (11.76)	0.97	19 (15.32)	7 (7)	0.05
Mode of exit = >Death	4 (2.52)	6 (8.11)	0.05	2 (1.59)	8 (7.41)	0.028
Exit toward reeducation department	50 (31.45)	22 (29.73)	0.79	40 (31.75)	33 (30.56)	0.84
Transfer toward another hospital/ward	11 (6.92)	5 (6.76)	0.96	11 (8.73)	5 (4.63)	0.70
Discharged toward home	94 (59.11)	41 (55.41)	0.59	73 (57.93)	62 (57.41)	0.94
Deceased at 12 months	7 (4.4)	8 (10.8)	0.06	6 (4.76)	10 (9.26)	0.17

* *For one patient the EPICES score was missing*.

#### Demographic Characteristics

Overall, 234 patients with ischemic stroke were included in the three centers in French Guiana. Overall, 68.2% were considered precarious (Table 2). Over half of the population (52.49%) was born abroad. The main origins were Haiti 41.77%, Suriname 22.15%, Brazil 10.76%, Guyana 5.70%, and Saint Lucia 5.06%. Among precarious patients the proportion of foreign citizens was twice the proportion of French nationals (61.08 vs. 30.43%, respectively *P* < 0.0001). Precarious patients were younger than non-precarious (patients 60.77 years, SD: 14.5 vs. 64.4 years, SD: 14.29), *p* = 0.04. Foreign patients were younger than French patients (59.9 years, SD: 13.98 vs. 63.85 years, SD: 14.93), *p* = 0.01.

#### Stroke Mechanism

There was no significant difference in terms of ischemic stroke mechanism between precarious and non-precarious, and between foreign and French patients (Table 3).

#### Risk Factors

There was no significant difference between socially precarious and non-precarious patients, and between French and Foreign patients for any cardiovascular risk factors (Table 3). For cardiovascular medical history, there was no significant difference between precarious and non-precarious patients (Table 3). However, when comparing French and foreign patients, French patients were significantly more likely to have a history of lower limb arteriopathy and a history of vascular surgery than foreign patients.

Socially precarious patients were more likely to have a pre-existing disability than non-precarious patients (Table 3). No such difference was observed when comparing French and Foreign patients.

#### Treatment Before the Stroke

There was no significant difference between socially precarious and non-precarious patients in terms of the following treatments: anti-clotting drugs, anti-hypertensives, statins, insulin, oral anti-diabetics, and psychotropic drugs. French patients were more likely to have been on platelet anti-aggregant therapy before the stroke than foreign patients (Table 3).

#### Delays in Accessing Care

There was no significant difference between socially precarious and non-precarious patients in terms of delay between symptoms and admission, delay between admission and thrombolysis, and in the proportion of patients benefiting from thrombolysis (Table 3). Similarly, there was no significant difference between foreign and French patients in terms of symptoms to admission interval or in the proportion of patients benefiting from thrombolysis. However, French patients benefited from a significantly shorter delay between admission and thrombolysis than foreign patients (Table 3).

#### Paraclinical Examinations

Socially precarious patients were less likely to benefit from a first intention MRI than non-precarious patients (Table 3). These differences remained significant after adjusting for age. There was no other difference for any other explorations. Foreign patients were also less likely to benefit from a first intention MRI and from Supra-aortic and trans-cranial Doppler and ultrasonography than French patients (Table 3). These differences remained significant after adjusting for age. There was no other difference between French and foreign patients for other explorations.

#### Severity of Stroke and Case Fatality

Upon admission to hospital, there was no difference regarding the severity of stroke (NIH severity of 10 and above) between foreign and French patients and between precarious and non-precarious patients (Table 3). This remained so despite adjustments for age. However, when looking at exit mode (Table 3), socially precarious patients were less likely to die. Similarly foreign patients were significantly less likely to die than French patients (Table 3). After adjusting for age, the trend persisted but the differences were no longer significant AOR = 0.25, 95% CI = 0.05–1.2, *P* = 0.078 for precariousness and AOR = 0.23, 95% CI = 0.04–1.2, *P* = 0.095 for being a foreigner. Within 12 months after admission, in crude analyses (Table 3) and after adjustment for age, there was also a non-significant trend toward lower mortality among precarious and among foreign patients.

#### Destination at Discharge

There was no significant difference between socially precarious or non-precarious patients, or between foreign and French patients in terms of discharge mode (Table 3).

## Discussion

The comparison of ischemic stroke patient characteristics and outcomes between 2 contrasted French territories is important to pinpoint specific areas of intervention. Indeed, the characteristics of patients included in French Guiana were very different from those included in Dijon. Patients included in French Guiana were more precarious (EPICES score >30) than patients included in Dijon. Patients in French Guiana had a more precarious family situation; they were less often followed medically by general practitioners, specialists, and dentists. They were also less likely to have health insurance than in Dijon. The use of thrombolysis appeared less frequent in the patients included in French Guiana than in the patients included in Dijon but the difference disappeared after adjusting for patient characteristics.

Within French Guiana, there were also significant health inequalities which were more salient when comparing foreign patients with French patients than when comparing precarious patients using the EPICES score. Among stroke patients in French Guiana, Foreign patients were about 4 years younger than French patients. There was no significant difference between precarious and non-precarious patients regarding the delay between symptoms and admission, but among those who benefited from thrombolysis, French patients had a slightly shorter delay between admission and thrombolysis. Non-precarious patients were also more likely to benefit from first intention MRI than precarious patients. A counterintuitive finding was the lower 1-month mortality in socially precarious patients than in non-precarious patients, however the trend was no longer significant after adjusting for age and initial severity. This is different of what was observed in the UK where foreign patients generally have had a consistently higher mortality than UK citizens (19, 20). One of the hypotheses to explain this lower case-fatality is that socially precarious and foreign patients were younger and had fewer comorbidities, which improved survival rates despite apparently more severe strokes. Recently, it was shown that socially precarious patients had increased mortality which was delayed between 3 and 12 months; but here again this was not observed, perhaps within French Guiana the total number of deaths was insufficient for robust statistical analyses (21).

The study design did not include all patients with a stroke and thus did not allow calculating incidence. Over half of patients were foreign, which seems high and would suggest a higher incidence among foreign patients, but this corresponds to the demographic situation among adults in the general population of French Guiana (15). Over 2/3rds of the patients were considered socially precarious whereas in Dijon France it was 39.4%. Foreign patients were more frequently socially precarious than French-born patients. In French Guiana, 53% of persons live under the French National poverty level (4) and a study in 2011 showed that 73.3% of hospital patients in Cayenne were socially precarious (EPICES score>30). The proportion of socially precarious persons among stroke patients was high in French Guiana but it is difficult to affirm that the incidence of stroke was higher in precarious persons than in non-precarious persons; however the younger age at diagnosis would definitely suggest so. An important potential limitation in the present study is that the strict inclusion criteria requiring the presence of kin for patients with severe strokes and the requirement of reachability after discharge may have led to the exclusion of very precarious patients coming from the most remote areas of French Guiana, often late and alone. Not including these patients may have introduced a bias minimizing the representation of severe precarious patients consulting late. Future prospective studies in French Guiana should therefore have broader inclusion criteria to limit this bias. Patient follow-up by phone may have biased estimations as the availability of a phone may be less frequent in the precarious. Another limitation is that the Dijon Comprehensive Stroke Center may concentrate large artery occlusions that are referred from other smaller hospitals, which may partly explain the observed differences in mechanisms (more cardio-embolic etiology) and severity (greater NIHSS score on admission). Another potential limitation is that the comparisons within French Guiana may have required more statistical power to unmask differences between groups. Nevertheless, the present study was prospective and gives the first rigorous comparison of ischemic stroke between French Guiana and Dijon in France and the first study of health inequalities and stroke within French Guiana.

We have shown that among precarious populations, and notably among migrants, the picture for many diseases is often similar: a later presentation with more advanced disease and often more deaths (8–10, 22). Socially vulnerable populations will renounce care for reasons pertaining to cost or transport (11, 12, 16). For reasons that are unclear, the more socially vulnerable patients and foreign-born seemed less likely to benefit from MRI both in 1st intention and for control. Foreign-born patients were less likely to benefit from supra-aortic and transcranial doppler and ultrasonography. Foreign-born patients also had longer intervals between admission and thrombolysis but not between stroke and thrombolysis. The interval between stroke and admission seemed longer but this was not statistically significant. Previous studies in other countries have also shown some social inequalities in the provision of care notably regarding delays, imagery, rehabilitation treatment and prevention, but there was no consistent pattern of inequality by socioeconomic status (2). Here, one plausible explanation could be that uncertainties linked to the frequent linguistic barriers among foreign patients require longer intervals to ensure patients do not have contraindications for MRI or thrombolysis.

Overall, foreign and more socially vulnerable patients were younger and had more severe lesions but they were less likely to have a past vascular history. Despite some elements suggesting decreased access to care and diagnostics they had increased survival and were generally oriented in the same manner. The overall picture is thus nuanced. There seem to be social inequalities of health regarding stroke, but not quite to a level observed elsewhere, because patient survival was higher among the most socially vulnerable, despite more severe lesions, presumably because they were younger. This difference with observations from elsewhere (2) may reflect particularities in the demographics of immigrants in French Guiana; it could also show that hospital care is relatively accessible for the most vulnerable (11, 12) in the universal health care system in French Guiana. In line with theoretical work on the strength of weak ties and structural holes, ego network studies suggested that social networks topology also had an influence on delays to consult (23). Here we did not find any differences in pre-hospital delays between precarious and non-precarious groups, perhaps the small size of the cities and limited alternatives makes the health system easier to grasp in case of emergency. However, for prevention, screening, non-urgent care and chronic diseases such as hypertension or diabetes social vulnerability may definitely lead to dramatic outcomes. Three quarters of stroke risk factors are behavioral and thus amenable to prevention (1). Proactive interventions have demonstrated potential for rapid impact, for instance the US Hypertension Detection and Follow-up Project 1976–1979 rapidly lowered cardiovascular deaths by 26% (24). In the French territory with the highest incidence of stroke (6), this suggests that health promotion and screening efforts should be made to reach the population, and notably the most vulnerable groups, with rapid potential gains in terms of stroke-related morbidity and mortality. Beyond social inequalities in identifying the proximal determinants of stroke, studies have shown that the buildup toward these inequalities starts early in life with childhood circumstances, nutritional deficiencies during pregnancy and infancy (2, 25). In the territory with the highest fertility rate in Latin America, and one of the highest preterm delivery rate (9), where 30% of the population is below the poverty level, improvements in mother and child care may lead to additional benefits with regards to stroke. Moreover, lead, which has been listed among the risk factors for stroke (1) is at concentrations above 50 micrograms among 25% pregnant women (26) and the awareness of the magnitude of this problem, which predominantly affects poorer populations, is only dawning on health authorities. What impact this could have on the risk of stroke among the more socially vulnerable remains to be determined.

In conclusion, within French Guiana, over 2/3rds of patients were socially precarious and over half were foreigners; the differences between socially precarious and non-precarious patients were not as important as described elsewhere; the main difference was that foreigners were younger than French patients at the time of their stroke. Ischemic stroke in French Guiana was strikingly different from ischemic stroke in Dijon: patients were substantially younger and poorer in French Guiana than in Dijon; strokes were predominantly lacunar in French Guianaand cardio-embolic in Dijon but this may have reflected referrals of patients with large artery obstruction to the large reference stroke center in Dijon. Although thrombolysis was substantially more frequent in Dijon than in French Guiana in the crude analysis, taking into account patient differences showed no difference in the proportion of thrombolysis between French Guiana and Dijon. Within French Guiana, the risk factors, mechanisms, and outcomes seemed relatively homogenous across socioeconomic strata. Therefore, the marked differences between 2 contrasted French territories suggest that beyond health inequalities there may be a different epidemiology of cardiovascular risk factors between French Guiana and mainland France.

## Data Availability Statement

The raw data supporting the conclusions of this article will be made available by the authors, upon reasonable request.

## Ethics Statement

The cohort was approved by the French Regulatory authorities CNIL (Commission Nationale de l'Informatique et des Libertés, autorisation n°911040) and the CPP Est 1/AFSSAPS (Comité de protection des personnes Est 1, Agence National de Sécurité du médicament et des Produits de Santé, autorisation n°2010/40 – AFSSAPS n°2010-A00940-39). All patients or legal representatives gave consent to participate. The patients/participants provided their written informed consent to participate in this study.

## Author Contributions

DR, MN, IF, and YB: conception. DR, EM, CM, MP-P, IF, YB, and ED: study conduct and inclusions. DR and IF: data analysis. MN: first draft writing. DR, CM, BdT, NS, IF, and MN: review and editing. All authors contributed to the article and approved the submitted version.

## Funding

The study was funded by the PHRC (Programme Hospitalier de Recherche Clinique), French Ministry of Health.

## Conflict of Interest

The authors declare that the research was conducted in the absence of any commercial or financial relationships that could be construed as a potential conflict of interest.

## Publisher's Note

All claims expressed in this article are solely those of the authors and do not necessarily represent those of their affiliated organizations, or those of the publisher, the editors and the reviewers. Any product that may be evaluated in this article, or claim that may be made by its manufacturer, is not guaranteed or endorsed by the publisher.
